# False Positivity of Anti-SARS-CoV-2 Antibodies in Patients with Acute Tropical Diseases in Thailand

**DOI:** 10.3390/tropicalmed7070132

**Published:** 2022-07-12

**Authors:** Viravarn Luvira, Pornsawan Leaungwutiwong, Narin Thippornchai, Charin Thawornkuno, Supawat Chatchen, Wiwat Chancharoenthana, Sarunporn Tandhavanant, Sant Muangnoicharoen, Watcharapong Piyaphanee, Narisara Chantratita

**Affiliations:** 1 Department of Clinical Tropical Medicine, Faculty of Tropical Medicine, Mahidol University, Bangkok 10400, Thailand; viravarn.luv@mahidol.ac.th (V.L.); wiwat.cha@mahidol.ac.th (W.C.); sant.mua@mahidol.ac.th (S.M.); watcharapong.piy@mahidol.ac.th (W.P.); 2 Department of Microbiology and Immunology, Faculty of Tropical Medicine, Mahidol University, Bangkok 10400, Thailand; narin.thp@mahidol.ac.th (N.T.); sarunporn.tan@mahidol.ac.th (S.T.); narisara.cha@mahidol.ac.th (N.C.); 3 Department of Molecular Tropical Medicine and Genetics, Faculty of Tropical Medicine, Mahidol University, Bangkok 10400, Thailand; charin.tha@mahidol.ac.th; 4 Department of Tropical Pediatrics, Faculty of Tropical Medicine, Mahidol University, Bangkok 10400, Thailand; supawat.cht@mahidol.ac.th; 5 Mahidol-Oxford Tropical Medicine Research Unit, Faculty of Tropical Medicine, Mahidol University, Bangkok 10400, Thailand

**Keywords:** COVID-19, SARS-CoV-2, dengue, ELISA, acute febrile illness, false positive reaction, cross reaction, tropical diseases, antibodies, Thailand

## Abstract

Serology remains a useful indirect method of diagnosing tropical diseases, especially in dengue infection. However, the current literature regarding cross-reactivity between SARS-CoV-2 and dengue serology is limited and revealed conflicting results. As a means to uncover relevant serological insight involving antibody classes against SARS-CoV-2 and cross-reactivity, anti-SARS-CoV-2 IgA, IgM, and IgG ELISA, based on spike and nucleocapsid proteins, were selected for a fever-presenting tropical disease patient investigation. The study was conducted at the Faculty of Tropical Medicine during March to December 2021. The study data source comprised (i) 170 non-COVID-19 sera from 140 adults and children presenting with acute undifferentiated febrile illness and 30 healthy volunteers, and (ii) 31 COVID-19 sera from 17 RT-PCR-confirmed COVID-19 patients. Among 170 non-COVID-19 samples, 27 were false positives (15.9%), of which IgA, IgM, and IgG cross-reactive antibody classes were detected in 18 (10.6%), 9 (5.3%), and 3 (1.8%) cases, respectively. Interestingly, one case exhibited both IgA and IgM false positivity, while two cases exhibited both IgA and IgG false positivity. The false positivity rate in anti-SARS-CoV-2 IgA and IgM was reported in adults with dengue infection (11.3% and 5%) and adults with other tropical diseases (16.7% and 13.3%). The urea dissociation method applied to mitigate false positivity resulted in significantly decreased ELISA-based false and true positives. In conclusion, the analysis of antibody against SARS-CoV-2 in sera of patients with different tropical diseases showed that high IgA and IgM false positivity thus potentially limits serological assay utility in fever-presenting patients in tropical areas.

## 1. Introduction

An outbreak of coronavirus disease 2019 (COVID-19), due to infection with severe acute respiratory syndrome coronavirus 2 (SARS-CoV-2), was identified in December 2019 in Wuhan, China, and has been classified as a global pandemic since March 2021 [1]. Polymerase chain reaction (PCR) remains the gold standard for COVID-19 diagnosis. The viral loads detected in patients are high during the first week post-symptom onset and gradually decline with time thereafter [2]. The potential benefits of serology in diagnosing COVID-19 are (i) in identifying PCR-negative COVID-19 cases, particularly in patients presenting in the later stages of disease progression with low viral load such as multisystem inflammatory syndrome in children (MIS-C); (ii) epidemiologic studies; and (iii) vaccine studies [3]. There are a number of commercial serological testing methods, both enzyme-linked immunosorbent assay (ELISA) and point-of-care (POC), available with variations in sensitivity, specificity, and accuracy [4,5]. However, the limitations of serology are false positives and false negatives. Theoretically, false negative COVID-19 serology results may occur in the early phase of infection, especially in mild cases and with application of low sensitivity serological techniques. False positive serology results for COVID-19 can primarily be attributed to cross-reactivity with other coronaviruses [6,7] or endogenous proteins in sera such as well-documented rheumatoid factor (RF) and antinuclear antibodies (ANA) [8,9,10]. Urea dissociation, based on the dissociation of low-avidity antibodies caused by a substance, such as hypermolar solutions of urea [11], that disrupts hydrogen bonds, was previously reported to successfully resolve cross-reactivity from RF, minimizing the risk of false positive results of IgM and IgG antibodies against SARS-CoV -2 in many studies [10,12,13].

False positive dengue IgM from POC tests in confirmed COVID-19 cases was also reported [14]. However, information regarding cross-reactivity between SARS-CoV-2 and dengue serology is limited and revealed conflicting results [15,16,17,18]. A previous study revealed up to 21.8% false positive/equivocal results from anti-SARS-CoV-2 IgA/IgG by ELISA testing in dengue samples [15], while other studies reported minimal false positive anti-SARS-CoV-2 when using the POC test in sera of dengue patients [16,17]. The cross-reactivity of tropical diseases, such as dengue, with COVID-19 has been an issue of concern in tropical areas. Furthermore, the serological cross-reactivity of zika virus with COVID-19 has also been reported [19]. 

Fever and non-specific symptoms (e.g., myalgia, diarrhea, and rash) of COVID-19 make it difficult to distinguish from other tropical infectious diseases, particularly dengue infection [20,21]. The common tropical diseases causing acute undifferentiated febrile illness (AUFI) in urban settings in Thailand were dengue (39.6%), follow by murine typhus (5%), leptospirosis (3.5%), and influenza (1.5%) [22]. Inevitably, serology remains an important diagnostic testing tool of tropical diseases [23]. It is currently unclear whether common tropical diseases such as dengue, rickettsiosis, influenza, and leptospirosis provide false positives in ELISA based on spike and nucleocapsid proteins of SARS-CoV-2 [15,16,17,18]. Therefore, in this study, we aimed to analyze the cross-reactivity among different classes of antibodies against SARS-CoV-2 proteins using archived sera from patients with common tropical diseases collected before the COVID-19 pandemic. The information of cross-reactivity between tropical infections and COVID-19 will provide benefits for diagnostic measures and preventative treatment in early infections. 

## 2. Materials and Methods

### 2.1. Serum Samples

In order to evaluate the cross-reactivity of COVID-19 and tropical diseases causing AUFI, the study sample size was calculated based on the previously reported false positivity rate of 21.8% [15]. The calculated sample size of 100 was used for the non-COVID-19 samples (true negative samples) [24].

**Figure 1:**
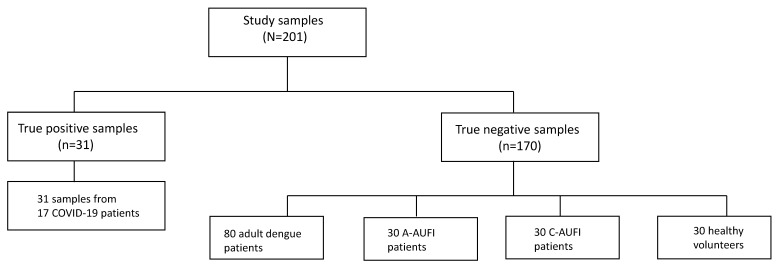
Serum samples used in the study. A-AUFI, adults with undifferentiated febrile illness; C-AUFI, children with acute undifferentiated febrile illness.

True positive samples were collected from COVID-19 patients admitted to the Hospital for Tropical Diseases (HTD), Faculty of Tropical Medicine, Mahidol University, Bangkok, Thailand during the first wave of COVID-19 outbreak in Thailand in 2020 [25]. (Figure 1) The diagnosis was confirmed by real-time reverse transcription PCR (RT-PCR) (detection kit for novel coronavirus 2019-nCoV RNA; DaAn Gene Co., Ltd., Guangzhou, China) from nasopharyngeal swab and throat swab. There were 31 COVID-19 sera from 17 patients. The acute sera were collected from 16 patients within the first week of onset (≤7 days). The convalescent serum samples were collected from 15 patients after the first week of onset (>7 days). True negative specimens consisted of four groups of archived specimens collected before the COVID-19 era as follows: adult dengue, adult with other tropical diseases causing AUFI (A-AUFI group), children with AUFI (C-AUFI group), and healthy individuals (Figure 1). The convalescent serum adult dengue and adult AUFI samples were selected from the previous cohort enrolled at the HTD during 2013–2015 [22], consisting of patients diagnosed with dengue fever (n = 80), influenza (n = 10), murine typhus (n = 10), and leptospirosis (n = 10), and were used to test the specificity and cross-reactivity of the SARS-CoV-2 serology. Thirty convalescent samples from children with AUFI were from the cohort at Ratchaburi Provincial Hospital, Ratchaburi, during 2006–2009 [26]. In principal, the diagnosis of dengue infection and other tropical infections was based on positive PCR and/or seroconversion of standard serology, as previously described [22,26]. In cases where the pathogen was not identified by PCR and serology, clinical diagnosis was applied. Thirty healthy sample were collected from healthy individuals at Udon Thani Hospital, Udon Thani between August 2018 and August 2019.

### 2.2. ELISA Assays

Anti-SARS-CoV-2 assay is an ELISA that determines the human antibodies of the immunoglobulin classes IgG, IgA, and IgM against SARS-CoV-2 in serum or plasma sample by semiquantitative in vitro determination (EUROIMMUN, Lübeck, Germany) [27]. The IgG and IgA microplates were coated with S1 domains of the SARS-CoV-2 spike protein, while the IgM microplate was coated with modified nucleocapsid protein (NCP) of SARS-CoV-2. The specific anti-SARS-CoV-2 immunoglobulins were attached to the antigens through incubation with diluted patient serum samples at a ratio of 1:100 in sample buffer, which was provided by the company. The microplate was then washed to remove all non-specific binding; the antibodies were detected by adding an enzyme conjugate containing anti-human IgG, IgA, or IgM labelled with peroxidase. Subsequently, a chromogen or peroxidase substrate tetramethylbenzidine (TMB) was added to develop a blue color. Finally, the reaction was stopped by adding 0.5 M sulfuric acid and the photometric measurement was performed at a wavelength of 450 nm. The optical density (OD) indicated the quantity of the specific anti-SARS-CoV-2 antibodies contained in samples. The results were evaluated semiquantitatively by calculating a ratio of the extinction of the patient serum sample over the extinction of the calibrator. This ratio was interpreted as follows: <0.8 negative; ≥0.8 to <1.1 borderline, and ≥1.1 positive [27]. 

### 2.3. Urea Dissociation Test

In this study, we tested whether the dissociation of urea can reduce false positives in ELISA. The dissociation of urea was performed as previously described [12,13]. In brief, the IgG and IgA microplates were coated with S1 domain of the SARS-CoV-2 spike protein, while the IgM microplates were coated with modified SARS-CoV-2 NCP. Upon adding sera, the microplates were washed to remove all non-specific binding, and the urea dissociation was performed by adding 100 µL of PBS solution containing 4 mol/L of urea and incubated at 37 °C for 10 min. After washing, the antibodies were detected by adding an enzyme conjugate containing either anti-human IgG, IgA, or IgM. The substrate solution was added and the reaction was stopped by adding 0.5 M sulfuric acid. The measurement was performed at a wavelength of 450 nm. The results were evaluated semiquantitatively, as described previously. 

### 2.4. Ethics Statement

This study was approved by the Ethics Committee of the Faculty of Tropical Medicine, Mahidol University (MUTM 2020-043-01). The informed consent was waived. Participants signed consent with their original cohorts to allow leftover specimen usage for related studies. 

### 2.5. Statistical Analysis

*p*-values < 0.05 were considered statistically significant. Statistical analysis was performed using GraphPad Prism version 9.0 (GraphPad Software Inc., La Jolla, CA, USA). One-way ANOVA was used for comparing the mean of antibody levels between groups [28]. The sensitivity and specificity were calculated using the standard formula: sensitivity = true positive/(true positive + false negative) × 100; specificity = true negative/(true negative + false positive) × 100.

## 3. Results

### 3.1. Characteristics of COVID-19 and Acute Tropical Disease Patients

For COVID-19 patients, 31 samples, consisting of 16 acute and 15 convalescent sera, were collected from a total of 17 COVID-19 patients. All had mild to moderate symptoms with four diagnosed as pneumonia via symptomatic evaluation and chest radiography. 

The COVID-19 negative archived sera consisted of 80 adult dengue and 30 adult AUFI sera (10 influenza, 10 murine typhus, and 10 leptospirosis). The mean age was 33 ± 13.3 years; 55.5% were male. Among dengue cases, 64 were positive for both anti-dengue IgM and IgG, while 16 cases were positive for only anti-dengue IgG. Almost all (109 of 110) dengue and adult AUFI cases exhibited positive anti-dengue IgG.

To explore cross-reactivity, the children-AUFI group was included. This group consisted of 30 children who were ill from non-dengue AUFI and had negative dengue IgM and IgG. The mean age of this group was 8.4 ± 1.9 years. Clinical diagnoses were acute respiratory tract infections including pharyngitis, acute tonsillitis, and common cold.

### 3.2. False Positive, Sensitivity, and Specificity Anti-SARS-CoV-2 Serology 

**Figure 2:**
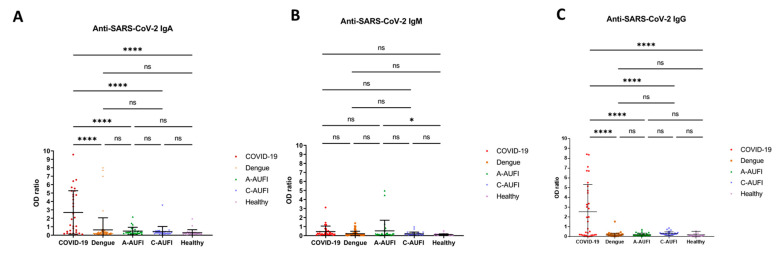
Anti-SARS-CoV-2 ELISA result distribution in each group. Analysis of anti-SARS-CoV-2 ELISA of IgA (**A**), IgM (**B**), and IgG (**C**) in serum samples from COVID-19, dengue, adults, and children patients with acute undifferentiated febrile illness (AUFI) and healthy persons. ns, non-significant; *, *p* < 0.05; ****, *p* < 0.0001.

Overall, there were 20 (64.5%), 4 (12.9%), and 17 (54.8%) true positives for anti-SARS-CoV-2 IgA, IgM, and IgG in COVID-19 samples, respectively. To evaluate false positivity, sensitivity, and specificity, borderline results (OD ratio 0.8–1.1) were considered as positive. False positivity was found in 27 out of 170 non COVID-19 cases (15.9%), consisting of 18 (10.6%), 9 (5.3%), and 3 (1.8%) cases corresponding to anti-SARS-CoV-2 IgA, IgM, and IgG, respectively. One case exhibited false positivity for both IgA and IgM and two cases exhibited false positivity for both IgA and IgG. Anti-SARS-CoV-2 ELISA results categorized into groups are plotted with the OD ratio in Figure 2. The rate of false positivity in each patient group is shown in Table 1 and the characteristics of borderline and false positive sera are described in Table 2. The high rate of false positivity in anti-SARS-CoV-2 IgA and IgM was found in adults with dengue infection (11.3% and 5%) and adult AUFI (16.7% and 13.3%). These suggested cross-reactivity between anti-SARS-CoV-2 antibodies and anti-dengue IgG rather than IgM, as almost all adult AUFI cases had positive anti-dengue IgG. 

The sensitivity and specificity of anti-SARS-CoV-2 antibodies were also calculated and are reported in Table 3. IgA showed the highest sensitivity (64.5%) with the lowest specificity (89.4%). IgG displayed the highest specificity (98.2%).

### 3.3. Anti-SARS-CoV-2 Serology after Urea Dissociation

To improve specificity of anti-SARS-CoV-2 IgM ELISA tests, the urea dissociation test with a urea concentration of 4 mol/L was applied as previously described [12,13].

**Figure 3:**
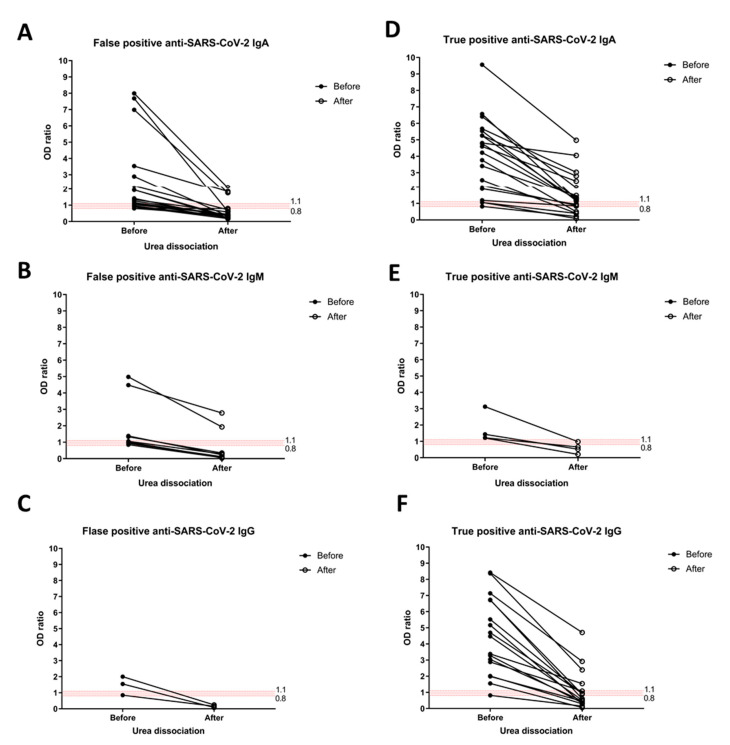
The anti-SARS-CoV-2 antibodies in correspondence with urea dissociation. Urea dissociation was performed with all borderline and positive results for anti-SARS-CoV-2 ELISA, both false positive (**A**–**C**) and true positive (**D**–**F**). The results of anti-SARS-CoV-2 IgA (**A**,**D**), IgM (**B**,**E**), and IgG (**C**,**F**) prior and subsequent to urea dissociation were plotted. An OD ratio <0.8 was considered as negative, 0.8–1.1 was considered as borderline (red horizontal area), and ≥1.1 was considered positive.

Overall, the test results showed a reduction in non-specific binding resulting in lower rates of false positivity in all anti-SARS-CoV-2 antibodies (Figure 3). There remained some false positives in 3 out of 18 anti-SARS-CoV-2 IgA and 2 out of 9 anti-SARS-CoV-2 IgM. However, this also decreased the reaction of true positive results. The true positive anti- SARS-CoV-2 IgA, IgM, and IgG (in confirmed COVID-19 cases) turned negative upon urea dissociation in 5 out of 20, 3 out of 4, and 9 out of 17 cases, respectively (Figure 3).

## 4. Discussion

We herein reported the high incidence of false positive anti-SARS-CoV-2 antibodies detected by ELISA, especially in the adult dengue and AUFI groups. The rate of false positivity was highest in IgA, followed by IgM, and lowest in IgG. Surprisingly, the rate of false positives was higher among adult dengue and adult AUFI patients than children with AUFI, suggesting cross-reactivity between dengue IgG antibodies to SARS-CoV-2. This occurrence is due to the high prevalence of previous dengue infection (positive anti-dengue IgG) among our adult participants, as previously reported in Thai adults [29]. We further explored the utility of the urea dissociation technique, which was previously successful in resolving cross-reactivity between autoantibodies, particularly RF and anti-SARS-CoV-2 antibodies [10,12,13]. However, it could not resolve all false positivity in our experiment, suggesting other cross-reactivity apart from RF. 

Nonspecific symptoms, including fever, myalgia, diarrhea, and rash, overlap symptoms of COVID-19 and other tropical diseases like dengue. Moreover, dengue and SARS-CoV-2 co-infection have been reported to cause more severe outcomes [30]. This challenging issue requires accurate and affordable diagnostic testing for tropical low-to-middle income countries. The serology for COVID-19 detection was applied in patient triage in some settings [31,32] and the serology is the main diagnostic method in tropical diseases [23]; therefore, cross-reactivity is a matter of concern that needs to be explored for patient triage policy making.

Our results reported a high rate of false positivity in anti-SARS-CoV-2 IgA and IgM ELISA in sera collected before the COVID-19 pandemic, thus suggesting cross-reactivity between dengue IgG and antibodies of SARS-CoV-2, similar to a previous study from Israel [15]. However, there was a low false positive rate with anti-SARS-CoV-2 IgG, similar to a previous study from Qatar [33]. Furthermore, we explored the utility of anti-SARS-CoV-2 ELISA in other tropical diseases apart from dengue. These results limit the utility of anti-SARS-CoV-2 IgA and IgM in patients suffering from fever in tropical areas. Thus, more specific serology tests and cut-off levels are required. A four-fold increase in titer or higher specific quantitative criteria for ELISA may overcome cross-reactivity limitations. However, this approach may cause tradeoffs in terms of decreased sensitivity. Serology by plaque reduction neutralization test (PRNT) could be used to distinguish between *Flaviviridae* and *Coronaviridae*. Further studies in order to improve the sensitivity and specificity of COVID-19 serology diagnosis are required. A combination of serology and direct diagnosis of SARS-CoV-2 (molecular method and antigen detection) may improve the sensitivity and specificity of COVID-19 detection. 

A broad knowledge of cross-reactivity between anti-dengue and anti-SARS-CoV-2 is worth exploring. A study from Brazil, a dengue endemic area, reported significant COVID-19 mortality among patients with a previous history of dengue infection [34]. A previous bioinformatics study showed structural similarities in envelope protein chains of dengue virus and SARS-CoV-2 spike protein [15]. Moreover, a study using computational modelling predicted with high confidence that dengue antibodies may bind to the receptor binding domain of SARS-CoV-2 spike protein [35]. 

The cross-reactivity of the SARS-CoV-2 serologic assays with other human coronaviruses could be part of the false positivity, as previously reported [6,36]. Unfortunately, the information of seroprevalence of other human coronaviruses within the general population in Thailand is limited. Furthermore, archived specimens in this study were not subject to other human coronaviruses testing. 

Cross-reactivity of RF with anti-SARS-CoV-2 antibodies is very important. Thus, the urea dissociation test was applied to solve false positivity of anti-SARS-CoV-2 from RF, as previously described [10,12,13]. The remaining false positives in this study may source from other cross-reactivity apart from RF, such as ANA [10], which is usually considerably greater in the older group than in the younger group [37]. This may contribute to a higher rate of false positives among adults than in children, as in our findings.

While other previous studies used specimens from only dengue fever and COVID-19 patients, we additionally explored false positivity among patients suffering from three other tropical infectious causes. Whereas previous studies reported only false positivity of anti-SARS-CoV-2 IgM and IgG, we further studied false positivity of anti-SARS-CoV-2 IgA. However, the limitation in variation of etiology might limit the generalization of the study results. Using single commercial ELISA may also limit the study’s implications, despite the brand being approved by international and national organizations. Moreover, the low sensitivity of anti-SARS-CoV-2 ELISA in this study may be the result of using a limited number of convalescent sera in COVID-19 patients for evaluation. Further study is warranted.

## 5. Conclusions

We report a high rate of false positivity in anti-SARS-CoV-2 IgA and IgM ELISA in dengue and other tropical diseases in Thailand, a tropical dengue epidemic country, but low false positivity rates in anti-SARS-CoV-2 IgG. The results may limit the utility of anti-SARS-CoV-2 IgA and IgM in clinical practice in tropical areas where dengue is co-epidemic, while anti-SARS-CoV-2 IgG may alternatively be used. More specific testing and cut-offs are required to improve the specificity of serology tests. Furthermore, in-depth mechanisms underlying cross-reactivity and COVID-19 pathophysiology in previous dengue-infected patients warrant further exploration.

## Figures and Tables

**Table 1:** False positivity rate of anti-SARS-CoV-2 ELISA in non-COVID-19 serum samples.

Group	N	False Positive Anti-SARS-CoV-2		
		IgA (%)	IgM (%)	IgG (%)
**Adult tropical diseases**	110	14 (12.7%)	8 (7.3%)	1 (0.9%)
**-Dengue**	80	9 (11.3%)	4 (5%)	1 (1.3%)
**-Adult-AUFI**	30	5 (16.7%)	4 (13.3%)	0
**Children with non-dengue AUFI**	30	2 (6.7%)	1 (3.3%)	1 (3.3%)
**Healthy**	30	2 (6.7%)	0	1 (3.3%)

**Table 2:** List of patients and healthy individuals with borderline and false positive anti-SARS-CoV-2 ELISA.

No.	Group	Diagnosis	Anti-Dengue		Anti-SARS-CoV-2		
			IgM	IgG	IgA	IgM	IgG
6	Adult AUFI	Dengue	positive	positive	borderline	negative	negative
19	Adult AUFI	Leptospirosis	negative	positive	negative	positive	negative
22	Adult AUFI	Influenza	negative	positive	positive	negative	negative
26	Adult AUFI	Dengue	positive	positive	negative	borderline	negative
32	Adult AUFI	Murine typhus	negative	positive	borderline	negative	negative
41	Adult AUFI	Dengue	negative	positive	positive	negative	negative
48	Adult AUFI	Dengue	negative	positive	negative	positive	negative
51	Adult AUFI	Dengue	positive	positive	positive	negative	negative
54	Adult AUFI	Leptospirosis	negative	positive	negative	positive	negative
59	Adult AUFI	Murine typhus	negative	positive	borderline	negative	negative
72	Adult AUFI	Dengue	positive	positive	positive	negative	negative
84	Adult AUFI	Dengue	negative	positive	positive	negative	negative
87	Adult AUFI	Dengue	positive	positive	positive	negative	negative
88	Adult AUFI	Murine typhus	negative	positive	negative	positive	negative
93	Adult AUFI	Dengue	positive	positive	negative	borderline	negative
94	Adult AUFI	Murine typhus	negative	positive	positive	borderline	negative
95	Adult AUFI	Dengue	positive	positive	negative	borderline	negative
97	Adult AUFI	Dengue	positive	positive	borderline	negative	negative
104	Adult AUFI	Dengue	positive	positive	positive	negative	positive
106	Adult AUFI	Murine typhus	negative	positive	positive	negative	negative
109	Adult AUFI	Dengue	positive	positive	positive	negative	negative
113	Children with AUFI	Bronchitis	negative	negative	negative	negative	borderline
115	Children with AUFI	Acute tonsillitis	negative	negative	negative	borderline	negative
130	Children with AUFI	Acute pharyngitis	negative	negative	borderline	negative	negative
132	Children with AUFI	Pharyngitis	negative	negative	positive	negative	negative
184	Healthy	Healthy	negative	positive	positive	negative	positive
187	Healthy	Healthy	negative	positive	positive	negative	negative

**Table 3:** Sensitivity and specificity of anti-SARS-CoV-2 ELISA.

Anti-SARS-CoV-2 ELISA	Sensitivity (%)	Specificity (%)
**IgA**	64.5	89.4
**IgM**	12.9	94.7
**IgG**	54.8	98.2

## Data Availability

The dataset can be requested from the corresponding author.
